# Prevalence of asystole during tilt test-induced vasovagal syncope may depend on test methodology

**DOI:** 10.1093/europace/euac154

**Published:** 2022-09-05

**Authors:** Vincenzo Russo, Erika Parente, Antonella Groppelli, Giulia Rivasi, Marco Tomaino, Alessio Gargaro, Daniele Giacopelli, Andrea Ungar, Gianfranco Parati, Artur Fedorowski, Richard Sutton, J Gert van Dijk, Michele Brignole

**Affiliations:** Chair of Cardiology, University of the Study of Campania ‘Luigi Vanvitelli’, Ospedale Monaldi, Via Leonardo Bianchi, 80131 Napoli, Italy; Chair of Cardiology, University of the Study of Campania ‘Luigi Vanvitelli’, Ospedale Monaldi, Via Leonardo Bianchi, 80131 Napoli, Italy; IRCCS Istituto Auxologico Italiano, Faint & Fall programme, Cardiology Unit and Department of Cardiology, S.Luca Hospital, Piazzale Brescia 2, 20149 Milan, Italy; Division of Geriatric and Intensive Care Medicine, University of Florence and Azienda Ospedaliero-Universitaria Careggi, Largo Brambilla 3, 50139 Florence, Italy; Ospedale Generale Regionale, Via Lorenz Böhler, 5, 39100 Bolzano, Italy; Research Clinical Unit, BIOTRONIK Italia S.p.A., Via Alessandro Volta 16, 20093 Cologno, Monzese, Italy; Research Clinical Unit, BIOTRONIK Italia S.p.A., Via Alessandro Volta 16, 20093 Cologno, Monzese, Italy; Division of Geriatric and Intensive Care Medicine, University of Florence and Azienda Ospedaliero-Universitaria Careggi, Largo Brambilla 3, 50139 Florence, Italy; IRCCS Istituto Auxologico Italiano, Faint & Fall programme, Cardiology Unit and Department of Cardiology, S.Luca Hospital, Piazzale Brescia 2, 20149 Milan, Italy; Department of Cardiology, Karolinska Institute, Nobels väg 6, 171 77 Solna, Stockholm, Sweden; Department of Medicine, Karolinska Institute, Nobels väg 6, 171 77 Solna, Stockholm, Sweden; National Heart and Lung Institute, Imperial College, Department of Cardiology, Hammersmith Hospital, Du Cane Road, London, W12 0HS, UK; Department of Neurology, Leiden University Medical Centre, PO Box 9600, 2300RC Leiden, The Netherlands; IRCCS Istituto Auxologico Italiano, Faint & Fall programme, Cardiology Unit and Department of Cardiology, S.Luca Hospital, Piazzale Brescia 2, 20149 Milan, Italy

**Keywords:** Syncope, Head-up tilt testing, Vasodepression, Cardioinhibition, Asystole, Cardiac pacing

## Abstract

This review addresses tilt-testing methodology by searching the literature which reports timing of asystole and loss of consciousness (LOC). Despite the Italian protocol being the most widely adopted, its stipulations are not always followed to the letter of the European Society of Cardiology guidelines. The discrepancies permit reassessment of the incidence of asystole when tilt-down is early, impending syncope, compared with late, established LOC. Asystole is uncommon with early tilt down and diminishes with increasing age. However, if LOC is established as test-end, asystole is more common, and it is age-independent. Thus, the implications are that asystole is commonly under-diagnosed by early tilt-down. The prevalence of asystolic responses observed using the Italian protocol with a rigorous tilt down time is numerically close to that observed during spontaneous attacks by electrocardiogram loop recorder. Recently, tilt-testing has been questioned as to its validity but, in selection of pacemaker therapy in older highly symptomatic vasovagal syncope patients, the occurrence of asystole has been shown to be an effective guide for treatment. The use of head-up tilt test as an indication for cardiac pacing therapy requires pursuing the test until complete LOC. This review offers explanations for the findings and their applicability to practice. A novel interpretation is offered to explain why pacing induced earlier may combat vasodepression by raising the heart rate when sufficient blood remains in the heart.

## Introduction

Head-up tilt test (HUTT) is an important diagnostic tool for patients with suspected reflex syncope, to be applied after the initial clinical assessment.^1^ An asystolic (>3 s) response during HUTT has been widely used for identification of older subjects (>40 years) who may benefit from pacemaker therapy.^2^ The occurrence of asystolic responses is specific for reflex syncope, being almost never observed in patients without syncope and rarely in patients with cardiac syncope.^3,4^ The recent European Society of Cardiology (ESC) guidelines for cardiac pacing give a strong class I level of evidence A indication for pacing in patients of >40 years with asystolic tilt response and severe recurrent reflex syncopes.^5^ Thus, demonstration of asystolic response during HUTT has become an important part of syncope management and selection of therapy.

The prevalence of asystolic responses during HUTT varies greatly from study to study. The prevalence seems to be influenced by several different factors, the most important ones being the methodology and practical execution of HUTT and the clinical features of the patients. The aim of this study was to review the literature, including some original data from our laboratories, and to propose solutions for clinical practice and direction of future research.

We hypothesized that asystolic HUTT responses are much more frequent than commonly thought if tilt down commences after syncope [complete loss of consciousness (LOC)] has begun.

## Different prevalence of cardioinhibitory syncope with asystole during head up tilt tests

### Prevalence of asystole according to tilt protocols

In a meta-analysis of 55 studies with a total of 4361 patients undergoing HUTT for suspected reflex syncope, the average overall positivity rate was 37% with passive tilt protocol, 60% for isoproterenol protocol, and 66% for nitroglycerine protocol.^6^

Asystolic responses were rarely observed with both ‘passive’ (i.e. without drug provocation) and isoproterenol tilt protocols. For example, owing to the low positivity rate of the passive protocol (Westminster protocol), asystolic responses were observed by Barón-Esquivias *et al*.^7^ in only 45 (4%) of 1124 patients who underwent HUTT, which corresponded to 17% of positive tests. A HUTT protocol of 15 min in the upright position without drugs followed by 15 min. after low dose isoproterenol^8^ resulted in a positivity rate of 73 (61%) in 120 patients with only 5 (7%) asystolic responses. Sra *et al*.^9^ reported 6 (8.5%) asystolic events in a group of 70 patients who had a positive response during 30 min passive followed by 15 min isoproterenol HUTT. Subsequently, the authors of the same group^10^ reported a prevalence of asystolic responses in 19 (9%) out of 209 patients who had a positive HUTT response. Thus, isoproterenol protocols are suboptimal when demonstration of asystolic response during HUTT is required for syncope management and selection of therapy.

Nitroglycerine protocols (passive + sublingual nitroglycerine) have achieved higher rates of asystolic responses among patients undergoing HUTT (see below). Thus, we were prompted to review the role of tilt down timing with nitroglycerine protocols. In *Table 1*, we show results of English-language publications noted on Medline with a population >100 patients affected by syncope who underwent nitroglycerine HUTT and in which the prevalence of asystolic responses was reported. The relative prevalence of cardioinhibitory forms among positive tests proved similar during the passive and the nitroglycerine phase.^13,25,26^ HUTT with nitroglycerine protocols showed great variability of asystolic responses, ranging from 5 to 29% of patients. Several factors influenced the prevalence. Age is probably the most important factor. In a large multicentre population of 5236 patients, asystole occurred in 18% of patients <50 years, but progressively decreased with age to reach 3% in patients older than 80 years.^25^ Cardioinhibitory forms seem to be much more frequent in younger patients with emotional triggers (24%) than in those with peripheral triggers (10%).^26^ Male gender, smoking, diuretic therapy and traumatic syncope have also been found to be associated with a propensity for asystolic syncope in a recent study by Russo *et al*.^24^ The cardioinhibitory reflex occurs late in the vasovagal cascade with asystole starting late, i.e. after patients had already lost consciousness, in one third of cases.^17^ This suggests that the prevalence of the asystolic response depends very strongly on the time patients are tilted down, relative to the ongoing haemodynamic events of syncope. We propose that asystole is often not demonstrated if patients are tilted down early, at presyncope.

**Table 1:** Cardioinhibitory asystolic (>3 s) responses in studies with >100 patients using nitroglycerine challenge

	Tilt-down time	Study population	Tilt-positive	Asystole >3 s n (% of total)	Asystole >3 s % of positive	Mean age	Males
**Tilt down before syncope**							
Galetta *et al.*^11^	Fall in BP >50 mmHg	380	326 (86%)	4 (1%)	1%	46 ± 7	56%
Carvalho *et al.*^12^	Presyncope	2263	1235 (55%)	149 (6.6%)	12%	37 ± 18	56%
Gemein *et al.*^13^	Presyncope	225	100 (44%)	13 (6%)	13%	49 ± 20	53%
Raviele *et al.*^14^	Vasovagal pattern	235	152 (65%)	20 (8%)	13%	52 ± 20	47%
Groppelli A, personal communication	Presyncope	na	101	na	10%	60 ± 19	46%
Prabhu *et al.*^15^	Vasovagal pattern	816	395 (48%)	60 (7.3%)	15%	49 (25–65)	68%
**Tilt down when spontaneous symptoms are reproduced**							
Kurbaan *et al.*^16^	Syncope or limiting symptoms	472	368 (78%)	89 (19%)	24%	165: ≤35 years; 169: 36–64 years; 171: ≥65 years	45%
Rivasi *et al.*^25^	Reproduction of spontaneous symptoms^a^	5236	3129 (60%)	516 (10%)	16%	60 ± 22	45%
Furukawa *et al.*^26^	Reproduction of spontaneous symptoms^a^	380	252 (66%)	56 (15%)	22%	62 ± 20	54%
**Tilt down at syncope onset**							
Del Rosso *et al.*^18^	Syncope onset	202	140 (69%)	38 (19%)	27%	49 ± 19	45%
Del Rosso *et al.*,^19^ subgroup ≤65 years	Syncope onset	324	177 (55%)	49 (15%)	28%	51 ± 12	45%
Foglia-Manzillo *et al.*^20^	Syncope onset	164	101 (63%)	31 (19%)	31%	13 ± 3	30%
Glockler *et al.*^21^	Syncope onset	306	245 (80%)	77 (25%)	31%	43 ± 20	46%
Zyśko *et al.*,^22^ tilt table down in 10–18 s	Syncope onset	587	381 (65%)	105 (18%)	28%	44 ± 18	35%
**Tilt down when loss of consciousness is established or with a prolonged tilt-down time**							
Zyśko *et al.*,^22^ tilt table down in 47 s	Syncope onset, but very slow table tilt down	645	396 (61%)	168 (26%)	42%	42 ± 18	35%
Van Dijk *et al.*^23^	Syncope confirmed by video-EEG (321 pts with presyncope only excluded)	674	163 (24%) had syncope	68 (10%)	42%	46 (25–62)	47%
Russo *et al.*^24^	Established loss of consciousness	1285	853 (66%)	368 (29%)	43%	45 ± 19	50%

BP, blood pressure; CI, cardioinhibition; EEG, electroencephalogram; na, not available.

In the case of symptomatic delayed orthostatic hypotension pattern, the test was interrupted before loss of consciousness.

### Prevalence of asystole by the timing of tilt down (nitroglycerine protocols)

In *Table 1*, we have divided studies according to the timing of tilt down. In 6 populations,^11–15^ the patient was tilted down before syncope, because of developing vasovagal patterns or presyncope; in these populations, syncope might have occurred, but it was not the declared endpoint of the protocol. Among patients with a positive test, the rate of asystolic forms ranged from 1 to 15%. In three populations^13,16,17^ the tilt table was tilted down as soon as induced symptoms were recognised by patients, aiming to reproduce those occurring spontaneously in previous episodes. The rate of occurrence of asystole was 16–24%. In 5 populations,^18–22^ the tilt table was tilted down at syncope onset, as suggested by ESC guidelines,^5^ although the precise way in which syncope was assessed was not stated. The rate of occurrence of asystole ranged from 27 to 31%. Finally, in 3 populations, the tilt table was tilted down when LOC was established using detailed criteria or with a prolonged tilt-down time (mechanically slow tilt-table return to supine^22^ see below). In one study,^24^ the tilt table was tilted down when complete LOC was assessed by an observer, indicated by lack of response to vocal stimuli, loss of muscle tone, and jerking movements, whichever occurred first. In the study of van Dijk *et al.*,^23^ presyncope was an exclusion criterion and syncope was assessed by means of clinical observation and video electroencephalogram (EEG). The patients with asystole were 42% of those with syncope. In a substudy of Zyśko *et al.* ^22^ the tilt table was tilted down at syncope onset, but the process of tilt-down was very prolonged (47 s) due to technical characteristics of the tilt table with the effect of prolonging the head-up period, resulting in an asystole proportion of 43%.

**Figure 1:**
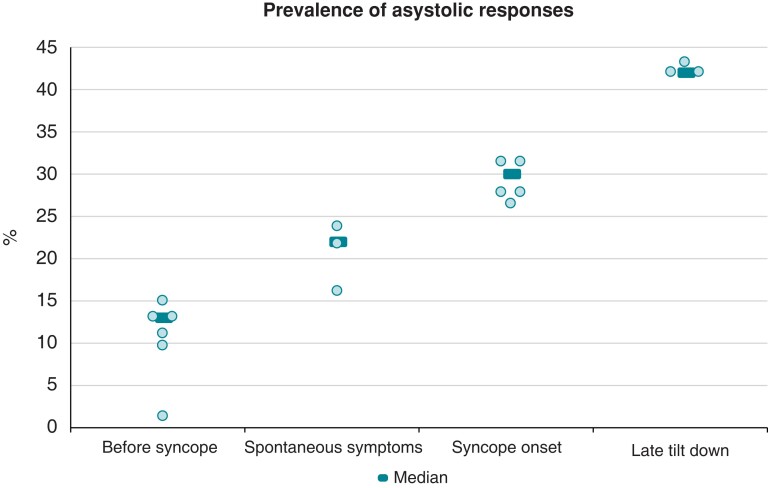
Cardioinhibitory asystolic (>3 s) responses in the four categories of populations subdivided by the presumed time of tilt down/resumption of supine position in the studies listed in the Table 1. Dark bars represent the median values for each category, and light bullets indicate the proportion of asystolic responses relative to positive tests of individual studies.

In the absence of documentation of the temporal relationship of asystole with the symptoms we may presume that the time to tilt down or the duration of tilt down were progressively longer through the above four categories (*Figure 1*). Further, the four categories suggest that prolongation of the upright position strongly influenced the type of response, with longer delays favouring the occurrence of asystole.

### The effect of age on asystolic responses

**Figure 2:**
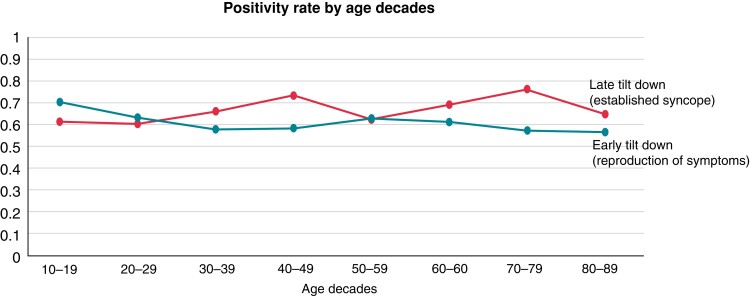
Overall positivity rate by age decades of HUTT in two large populations using the same tilt protocol, i.e. the Italian protocol, except for different timing of tilt down, one using *early tilt down* ^25^ and the other using a *late tilt down*.^24^

**Figure 3:**
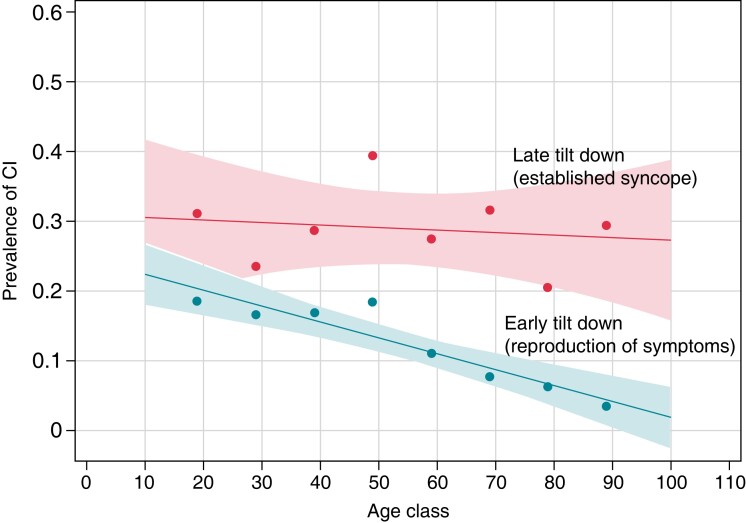
Comparison of the prevalence of positive asystolic forms between the same populations as *Figure 2*. The positivity rate is relative to the entire HUTT population including negative tests. The rate of asystolic forms decreased with advancing age (linear regression analysis, *P* = 0.001) in the study which used *early tilt* down,^13^ whereas it remained constant (*P* = 0.71) in the study with *late tilt down* ^11^. As a consequence, the difference in slopes between the two-line fits approached statistical significance (*P* = 0.079).

It is commonly accepted that the rate of occurrence of cardioinhibitory (including asystole) forms of syncope decreases with advancing age. In a large multicentre population of 5236 patients, asystole was present in 18% of patients <50 years and then progressively decreased to 3% in patients older than 80 years.^25^ The results of the population of Russo *et al*.^24^ are in contrast to the above data. We compared the two studies. They both applied the same HUTT protocol, namely the ‘Italian protocol,^27^ which consists of a 20 min passive phase at a tilt-angle of 60°, followed by a 15 min sublingual nitroglycerine phase (300–400 µg), if no syncope occurred during the passive phase. HUTT responses were classified according to the New VAso Vagal Syncope International Study (VASIS) classification.^28^ The study population was divided into age subgroups corresponding to age decades. Tilt testing-positivity rates and haemodynamic patterns of response were then analysed by age decades. Positive HUTT was defined as the reproduction of spontaneous symptoms accompanied by the characteristic vasovagal pattern of hypotension and bradycardia in the study of Rivasi *et al*.^25^ so this study represented ‘early tilt down’. In contrast, the study by Russo *et al*.^24^ represented ‘late tilt down’, as tilt down was based on confirmed LOC with lack of response to vocal stimuli, loss of muscle tone, and jerking movements, whichever occurred first.^24^ The overall positivity rate was similar in the two studies at all age decades (*Figure 2*). Conversely, the rate of asystolic forms decreased with advancing age (linear regression analysis, *P* = 0.001) in the study^25^ which used early tilt down timing, whereas it remained quite constant (*P* = 0.71) in the study with late tilt down timing (*Figure 3*).

Our interpretation of this unexpected finding is that the cardioinhibitory vagal reflex requires a longer time and a prolonged stressor to develop in older compared with younger patients, but, if these conditions are met, older patients may have a similar probability of developing asystolic syncope as younger patients, reflecting higher incidence of asystole recorded by implantable monitors. Thus, with a late tilt-down time, asystole appears to be determined mostly by the time to tilt down and not by the age of the patients.

## Comments on the above findings

### Correlation between spontaneous and tilt-induced syncope

It is commonly believed that an asystolic response during HUTT predicts that asystole also occurred during spontaneous syncope. However, the contrary is not the case: spontaneous asystolic syncope can be observed in patients with non-asystolic HUTT responses. In the ISSUE 3 trial,^29^ although asystolic response during HUTT was predictive of spontaneous ILR-recorded asystolic syncope in 86% of cases, an asystolic HUTT response was not observed in 52% of patients with asystolic spontaneous syncope. Early tilt down could explain why asystolic HUTT responses are more rarely observed during HUTT than during spontaneous syncope documented by implantable loop recorder (ILR). Indeed, when upright tilt position is maintained until complete LOC, the observed rate of asystolic syncope during HUTT was up to 43%, a figure which is not very different from the 52% rate of spontaneous asystolic episodes observed on ILR.^30^

### Timing of asystole and efficacy of cardiac pacing

‘Late asystole’, i.e. asystole starting ≤3 s before the onset of syncope or later^17^ raises concerns about the benefit of cardiac pacing. ‘Late asystole’occurred in one third of patients in a HUTT study using video-EEG to assess LOC,^17^ In that study, late asystole was considered unlikely to have been the primary cause of LOC; the authors suggested that this might explain the failure of cardiac pacing in some patients with reflex syncope.

Cardiac pacing is more likely to be effective in patients with early asystole, possibly because blood pressure is then still relatively high.^17^ Indeed, conventional back-up (hysteresis) pacing, starting when heart rate drops below 50 or 60 bpm, is unlikely to prevent LOC in patients with late asystole. In such patients, delaying the time of tilt down might result in asystole, but it could be questionable for the selection of patients who can benefit from cardiac pacing. However, this situation of late asystole is likely to occur when cardiac pacing is indicated in patients with spontaneous reflex asystolic pauses documented by ILR; indeed, in such circumstances, the temporal relationship between LOC and asystole cannot be assessed by ILR, nor can the contribution to syncope by vasodepression.

**Figure 4:**
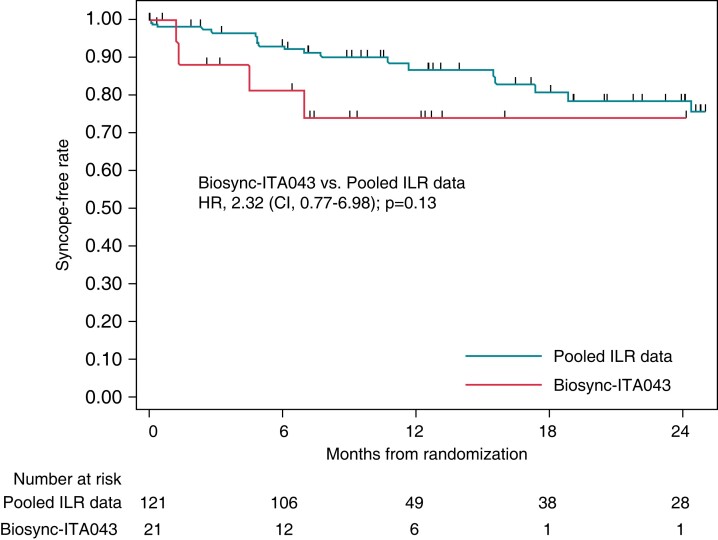
Comparison between syncope-free survival curves after cardiac pacing observed in a population of patients who had electrocardiographic documentation of asystolic syncope by means of implantable loop recorder (ILR)^28^ and those who had asystolic syncope diagnosed on HUTT with very late tilt down timing Biosync-ITA043 (Dr Russo’s centre).

In an ancillary analysis of the Biosync trial,^31^ the syncope-free survival curve of the 21 paced patients enrolled in the centre of Russo *et al*.^24^ (Biosync-ITA043), who used a late tilt down protocol, was compared with that of the 121 patients who were diagnosed by ILR in a metanalysis of 4 trials.^30^ The Kaplan Meier estimate showed a quite similar recurrence rate of syncope at 2 years of follow-up of 26 and 24% respectively (*Figure 4*). Russo’s subgroup rate was also quite similar to the rate observed in the overall population of Biosync trial, of 22% (not shown in *Figure 4*).

There are some potential explanations of the observed follow-up results:

1. Cardiac pacing is currently indicated in patients with electrocardiogram documentation of asystole in spontaneous reflex syncope. Lacking blood pressure data, this assessment ignores the relative contribution of vasodepression, which can be defined as all mechanisms decreasing blood pressure in reflex syncope that are not mediated by heart rate.^23^ This undetected vasodepression is the likely mechanism of post-pacing syncope recurrence. Some ILR patients undergoing pacemaker implantation are likely to have had late asystole similar to those observed in the HUTT population. At two years of follow-up, the recurrence rate of syncope with ILR was 24%,^30^ similar to 22% rate observed in the overall population of HUTT-induced asystole in the Biosync trial^31^ and to the 26% observed in Russo’s subgroup (*Figure 4*). This rate probably represents the real-world clinical scenario, in which heart rate data are available, but in which the relative amounts and timing of cardioinhibition and vasodepression cannot be assessed.
2. The role of cardioinhibition in reflex syncope should be re-examined. In a landmark study, van Dijk *et al.* ^23^ showed that, during HUTT induced reflex syncope, cardioinhibition (i.e. decrease in heart rate) started about 1 min (a median of 58 s) before LOC started. This heart rate decrease accounted for about half of the decrease in blood pressure at syncope, with vasodepression explaining the other half. Modern pacemakers designed to prevent reflex syncope have features allowing pacing at a programmable and relatively higher rate starting before asystole occurs, thus preventing the vagal reaction and slowing the fall in blood pressure, giving patients time to act.^9,32^

Moreover, the reproduction of the spontaneous vasovagal cascade during HUTT is only speculative and it is likely that some differences exist between spontaneous and induced vasovagal syncope. During HUTT, syncope occurs in the fixed upright position. Patients have no possibility to activate countermeasures to prevent the development of LOC. Conversely, during spontaneous episodes, patients may start fighting against reflex development and hypotension, assume sitting or supine position as soon as they recognise the symptoms of impending syncope. In this situation, hypotension is usually less severe and may be unable to cause complete LOC. The effects of severe bradycardia may be prevented by a pacemaker sustaining cardiac output by providing a higher heart rate.

This review has several limitations which arise from the non-uniform nature of the practice of the HUTT protocol, the heterogeneity in the demographic attributes of the study population, the heterogeneity of the mean age of the different studies and the lack of data regarding the contribution/timing of vasodepressor response to syncope even when asystolic response is present.

## Conclusions

The prevalence of cardioinhibitory responses during HUTT is underestimated and tilt down at the onset of syncope, as per ESC guidelines^5^ should be the norm. Asystolic HUTT responses are much more frequent, accounting for 27 to 31% of positive tests if the Italian protocol^6^ is performed as intended, meaning tilt down starts after the onset of syncope (complete LOC). The late tilt-down approach may also blunt the effect of ageing on the asystole rate. From a practical perspective, the use of HUTT as a therapy indication, especially for cardiac pacing, requires pursuing the test until the onset of syncope (complete LOC), as implied by ESC guidelines.^5^ The benefit of longer tilt times remains to be proven given the potential damage of prolonged ischaemia. However, if HUTT is used to confirm a diagnosis of vasovagal syncope, then the decision to tilt down may be based on complaint recognition, with additional instructions to minimise risk.^33^ Physicians should always mention in their HUTT report the protocol used and if tilt table was tilted down before or after syncope occurred.

## Data Availability

The data used for analysis are those reported in the Table 1.
